# Biosynthesis and characterization of a thermostable, alkali-tolerant chitinase from *Bacillus pumilus* JUBCH08 displaying antagonism against phytopathogenic *Fusariumoxysporum*

**DOI:** 10.1007/s13205-016-0406-x

**Published:** 2016-03-07

**Authors:** Sourav Bhattacharya, Arijit Das, Saikat Samadder, Subbaramiah Sundara Rajan

**Affiliations:** 1 Department of Microbiology, Center for Post Graduate Studies, Jain University, 18/3, 9th Main, 3rd Block, Jayanagar, Bangalore, 560011 Karnataka India; 2 Department of Microbiology, Centre for Advanced Studies in Biosciences, Jain University, Bangalore, 560019 Karnataka India

**Keywords:** *Bacillus pumilus*, Marine, Chitinase, Thermostable, Alkali-tolerant, Biocontrol

## Abstract

The present investigation highlights the process parameters influencing the submerged fermentation of chitinase by *Bacillus pumilus* JUBCH08, purification and characterization of the enzyme and determination of antagonistic activity of the bacterium against *Fusarium oxysporum*. Medium supplemented with 0.5 % chitin and peptone, at initial pH 8.0, when incubated at 35 °C for 72 h favored highest chitinase production. The enzyme was purified 25.1-fold to homogeneity. The chitinase was found to be thermostable and alkali-tolerant with maximum activity at pH 8.0 and 70 °C for 1 h. The molecular weight of chitinase was found to be 64 kDa by sodium dodecyl sulfate polyacrylamide gel electrophoresis. Mg^2+^, Co^2+^, Ca^2+^ and Mn^2+^ improved the chitinase activity. The *K*_*m*_ and *V*_max_ values of the enzyme were 0.13 mg/ml and 38.23 U/ml, respectively. When subjected to dual plate assay, the bacterium showed 45 % antagonism against *F. oxysporum*. Thus, it could be inferred that cultural conditions strongly affected the chitinase production by *B. pumilus* JUBCH08. The enzyme being thermostable and best functional under alkaline conditions could be useful for the feed industry and related biotechnological applications. Inhibition of *F. oxysporum* by the culture through lytic mechanism indicates its potentiality as a biocontrol agent.

## Introduction

Chitin, an insoluble polysaccharide, is one of the most abundant biopolymers on earth and is composed of linear chains of β-1, 4-*N*–acetyl glucosamine residues that are highly cross-linked by hydrogen bonds. Chitin as a structural polysaccharide is present in the fungal cell wall, nematodes, exoskeleton of insects and outer shell of crustaceans. Chitin forms a significant proportion of dry weight of the waste generated from shellfish industry (Wang and Chang 1997). Annually, shellfish production and processing industry contribute to the accumulation of a substantial amount of chitin rich waste that may prove to be an environmental hazard (Wang et al. 2002).

From the environmental point of view, chitin utilization is extremely essential for proper carbon–nitrogen balance. The utilization of chitinous wastes often involves corrosive chemicals resulting in high cost, low product yields and environmental toxicity. Chitinases (EC 3.2.1.14) are widespread in several bacteria, actinomycetes, fungi and higher plants (Swiontek Brzezinska et al. 2014).

Bacteria synthesize chitinase to breakdown chitin and metabolize it as carbon and energy sources (Lien et al. 2007). Numerous species of *Bacillus* had been reported to synthesize chitinase and act as biocontrol agents since they display antagonism against a wide range of plant pathogens that infect several economically important crops (Collins et al. 2003).

However, it should be noted that the use of chitinases is limited as the production and characteristics of chitinases often do not satisfy the industrial standards. Therefore, efforts must be directed toward finding newer sources of chitinases, better production strategies and ability of these chitinases to function under a wide range of temperature and pH. Studies on media optimization for chitinase production are a worthwhile approach because different media constituents may affect the production of microbial chitinases. Simultaneously, enzyme characterization is equally important to determine the optimal conditions under which the enzyme demonstrates its maximum activity. The present study aims at isolation and identification of a potent chitinolytic bacterium from marine sediment, optimized production, purification and characterization of chitinase and testing in vitro antagonistic activity of the bacterial isolate against phytopathogenic *Fusarium oxysporum*.

## Materials and methods

### Chemicals and reagents

All the analytical grade chemicals and media components used in the experiments were purchased from HiMedia, Mumbai, India.

### Source of fungal culture

*Fusarium oxysporum* MTCC 7678 was obtained from Microbial Type Culture Collection and Gene Bank (MTCC), Chandigarh, India. The fungus was propagated on potato dextrose agar (PDA) plates and stored at 4 °C until further used.

### Sampling

Bottom sediment for microbiological analysis was collected from the littoral zone (down to 5 cm) of Elliot’s Beach, Chennai Tamil Nadu, India (12.99°N 80.27°E). Following collection, sediment was aseptically transferred in a sterile zip-lock cover, stored in a container with ice and transported to the laboratory, where it was immediately processed (Donderski and Trzebiatowska 2000).

### Isolation of chitinase producing bacteria

Chitinase producing bacteria were isolated from the marine sediment sample using serial dilution and spread plate technique on mineral salt medium with the following composition (g/l) along with 1 % (w/v) colloidal chitin: (NH_4_)_2_SO_4_, 0.28; NH_4_Cl, 0.23; KH_2_PO_4_, 0.0067; MgSO_4_·7H_2_O, 0.04; CaCl_2_·2H_2_O, 0.022; FeCl_3_·6H_2_O, 0.005; yeast extract, 0.2; NaCl, 0.015; NaHCO_3_, 1.0 and 1 ml/l of trace element solution containing (g/l) ZnSO_4_·7H_2_O, 0.01; MnCl_2_·4H_2_O, 0.1; CuSO_4_·5H_2_O, 0.392; CoCl_2_·6H_2_O, 0.248; NaB_4_O_7_·10H_2_O, 0.177 and NiCl_2_·H_2_O, 0.02 with glucose (1 % w/v). The plates were incubated at 30 ± 2 °C for 5 days and following incubation examined for the appearance of bacterial colonies. The colonies showing clear zones against a cream background were considered as chitinase producers and maintained on chitin agar slants.

### Screening of chitinase producing bacteria

All the above isolates were individually inoculated in 100 ml of mineral salt broth supplemented with colloidal chitin (1 % w/v) and incubated at 30 ± 2 °C, 120 rpm for 5 days. Following incubation, the respective cultures were centrifuged at 10,000 rpm for 20 min at 4 °C. The cell-free supernatants were analyzed for enzyme activity (Kuddus and Ahmad 2013).

### Chitinase assay

The individual reaction mixture consisted of 1 ml of individual culture supernatant, 1 ml of 1 % (w/v) colloidal chitin in citrate phosphate buffer (0.1 M, pH 5.5) and incubated at 50 °C for 30 min. Following incubation, all the reaction mixtures were put in boiling water bath for a period of 2–3 min to stop the enzyme action. The solutions were centrifuged at 5000 rpm for 10 min. The amount of reducing sugar in the supernatants was determined by dinitrosalicylic acid method (Miller 1959). One enzyme unit was defined as the amount of enzyme that liberated 1 μmol of *N*-acetyl-D-glucosamine per minute under the standard assay conditions. The isolate demonstrating highest chitinase activity was selected for further study. The soluble protein contents of the enzyme samples were determined by Lowry’s method (Lowry et al. 1951).

### Molecular identification of selected bacterial isolate

The total genomic DNA was extracted from the bacterial cells using Bacterial Genomic DNA Isolation Kit (Chromous Biotech Pvt. Ltd., Bangalore, India) according to the manufacturer instructions and checked for its quality and concentration using 0.8 % (w/v) agarose gel electrophoresis.

16S rDNA region was amplified using universal forward 16S rDNA primer (5′-AGAGTTTGATCCTGGCTCAG-3′) and universal reverse 16S rDNA primer (5′-GGTTACCTTGTTACGACTT-3′). The PCR amplification was performed using the ABI 2720 thermal cycler (Applied Biosystems, USA) with 5 min initial denaturation at 94 °C, followed by 35 cycles that consisted of denaturation for 30 s at 94 °C, annealing for 30 s at 55 °C and extension at 72 °C for 1 min and a final extension of 5 min at 72 °C. Each reaction mixture contained 1 μl genomic DNA, 4 μl of dNTP’s (2.5 mM each); 10 μl of *Taq* DNA polymerase assay buffer and 1 μl *Taq* DNA polymerase (3 U/μl) (Chromous Biotech Pvt. Ltd., Bangalore, India) and the volume was made up to 100 μl using sterile distilled water. The PCR amplified product was eluted from the gel using Gel Extraction Spin-50 kit (Chromous Biotech Pvt. Ltd., Bangalore, India) according to the manufacturer instructions and detected by 1.2 % agarose gel (with ethidium bromide) electrophoresis.

The partial 16S rDNA sequencing of the PCR amplicon was performed at Chromous Biotech Pvt. Ltd., Bangalore, India using Big Dye Terminator Version 3.1 cycles sequencing kit and ABI 3500 XL Genetic Analyzer (Applied Biosystems, USA). The resultant nucleotide sequence was analyzed using the software Seq Scape version 5.2. The 16S rDNA sequence was aligned manually with the available nucleotide sequences retrieved from the NCBI database using BLASTn (Altschul et al. 1990). The nucleotide sequence was submitted to GenBank database (NCBI, USA) and was provided an accession number.

### Optimization of chitinase production

To determine the optimum concentration of colloidal chitin for chitinase production, the isolate was grown in the presence of different concentrations (0.1–2 % w/v) of colloidal chitin. Effect of nitrogen sources (0.1 % w/v: peptone, tryptone, yeast extract, beef extract, ammonium sulfate, urea and sodium nitrate) on chitinase production was studied. Physical parameters such as initial pH (4, 5, 6, 7, 8 and 9), incubation temperature (20, 25, 30, 35, 40, 45 and 50 °C) and incubation time (24, 48, 72, 96, 120, 144 and 168 h) were optimized for maximum chitinase production by the selected isolate.

### Enzyme purification

The cell-free supernatant of the chitinolytic isolate JUBCH08 was subjected to ammonium sulfate (70 % w/v) precipitation at 4 °C. The resulting precipitate obtained by centrifugation at 5000 rpm for 30 min at 4 °C was dissolved in phosphate buffer (0.2 M, pH 7.0), and dialyzed against the same buffer for 24 h. The dialyzed fraction was subjected to gel filtration chromatography using Sephadex G-200 column (1.5 × 10 cm) equilibrated with the phosphate buffer. Fractions were eluted with the same buffer at a flow rate of 1 ml/min. The fractions showing highest chitinase activity were pooled, concentrated and further purified using DEAE-Sepharose column (1.5 × 10 cm) pre-equilibrated with 10 mM Tris–HCl buffer (pH 7.6). The purified chitinase was eluted by a linear gradient of NaCl (0 to 0.5 M) in the same buffer. The flow rate was maintained at 0.5 ml/min. The active fractions having chitinase activity were collected, concentrated and used for characterization (Zhu et al. 2007). The molecular weight of purified chitinase was determined by SDS-PAGE (Laemmli 1970).

### Characterization of chitinase

The effect of pH on chitinase activity was evaluated using 0.1 M sodium acetate buffer (pH 3.0–6.0), 0.1 M Tris–HCl buffer (pH 7.0–9.0), and 0.1 M glycine-NaOH buffer (pH 10.0). pH stability of the enzyme was measured by incubating the enzyme at room temperature for 0–3 h in the buffer supporting the highest enzyme activity. The effect of temperature on chitinase activity was determined by exposing the reaction mixture at 10–100 °C at the optimum pH. Thermostability of the chitinase was assessed by incubating the enzyme for 0–3 h at the temperature supporting the highest activity. In both instances aliquots of the enzyme were collected at intervals of 30 min, cooled and the residual enzyme activity was determined as per standard assay procedure. Effect of various metal ions (chloridized Ca^2+^, Co^2+^, Mg^2+^, Hg^2+^, Ag^+^, Fe^3+^ and sulfated Cu^2+^, Ni^2+^, Zn^2+^, Mn^2+^) was determined by preincubation of the enzyme with 1 mM concentration of each metal salt at the optimum pH and temperature for 30 min. The Michaelis constant (*K*_*m*_) and the maximum velocity (*V*_max_) of the enzyme were determined from the double reciprocal plot by varying the colloidal chitin concentration from 0.2 to 2.0 mg/ml (Lineweaver and Burk 1934).

### Antagonistic activity against phytopathogenic *F. oxysporum* MTCC 7678

The fungal growth inhibition capacity of the selected bacterial isolate was determined by dual plate assay (Huang et al. 2005). Pure culture of *F. oxysporum* MTCC 7678 (5 mm agar plug) was placed at two opposite corners of PDA plate. The bacterial culture was inoculated at the center. Plates were incubated at 28 ± 2 °C for 5 days. Following incubation, growth diameter of the fungal pathogen was measured. In the control plate, the bacterial culture was replaced by sterile distilled water. Result was expressed as mean of the percentages of inhibition of *F. oxysporum* MTCC 7678 growth in the presence of the bacterial isolate.

Percentage inhibition was calculated using the following formula

$${\text{Percentage of inhibition}} = \, \left[ { 1- \left( {{\text{Fungal growth}}/{\text{Control growth}}} \right)} \right] \times 100$$

Mycelial morphology from the control and test plates was observed after staining with lactophenol cotton blue under binocular light microscope (Labomed, India).

### Statistical analysis

All the optimization studies were conducted in triplicate and the data were analyzed using single factor analysis of variance (ANOVA). All the data are graphically presented as mean ± SD of triplicates (*n* = 3). ANOVA was performed using Microsoft Excel 2007. *P* values < 0.05 were considered significant with a confidence limit of 95 %.

## Results and discussion

### Isolation and screening of chitinase producing bacteria

Twenty-one chitinolytic bacterial isolates were isolated from the marine sediment sample plated on mineral salt medium supplemented with 1.0 % w/v colloidal chitin. Among these, nine isolates showed chitinolytic zones corresponding to 15–20 mm. When these nine chitinolytic isolates were introduced in liquid medium, chitinase activity ranging between 6.8 and 13.1 U/ml was recorded. Isolate JUBCH08 which showed the highest enzyme production was selected for further studies.

### Molecular identification of the selected isolate

16S rDNA sequencing is a powerful tool for rapid identification and phylogenetic analysis of bacterial species. The apparent size of the PCR amplicon was ~1.5 kbp. The obtained 620 bp 16S rDNA nucleotide sequence was compared with available 16S ribosomal sequences in the NCBI database using BLASTn. The JUBCH08 isolate has been enrolled into a cluster containing *Bacillus* sp. and was found to be closely related to *Bacillus pumilus* strain XJ-117 with 99 % sequence similarity. Hence, it was designated as *Bacillus pumilus* JUBCH08. This nucleotide sequence submitted to GenBank was provided an accession number KC588945.

### Effect of colloidal chitin concentration

Since chitinase is an inducible enzyme, maximum chitinase is produced when the production medium contains optimum concentration of colloidal chitin as the substrate. Different colloidal chitin concentrations (0.1–2 % w/v) were investigated for chitinase production by *B. pumilus* JUBCH08, where the bacterial culture produced maximum chitinase (16.2 U/ml) when supplemented with 0.5 % (w/v) of colloidal chitin. Beyond this substrate concentration the enzyme activity decreased to reach a level of 8.3 U/ml with 2 % w/v of chitin in the medium.

A decline in chitinase production beyond 0.5 % (w/v) colloidal chitin concentration may indicate reduced utilization of colloidal chitin as substrate, or accumulation of intermediate metabolites produced from chitin decomposition, which might have inhibited the enzyme synthesis. According to the Michaelis–Menten equation, it may be hypothesized that the rate of an enzymatic reaction is directly proportional to the substrate concentration at a lower percentage. The greater the substrate concentration, the lesser would be its influence on microbial growth and rate of enzymatic reaction (Allison et al. 2014).

While solid-state cultivation of *B. thuringiensis* R 176 with shrimp shells and rice straw as a substrate for chitinase production was studied, the optimal medium contained 0.5 % (w/v) ball-milled chitin (Chaiharn et al. 2013). Thus, the present findings emphasize that chitinase production could be correlated with colloidal chitin concentration in the medium.

### Effect of nitrogen sources

**Fig. 1:**
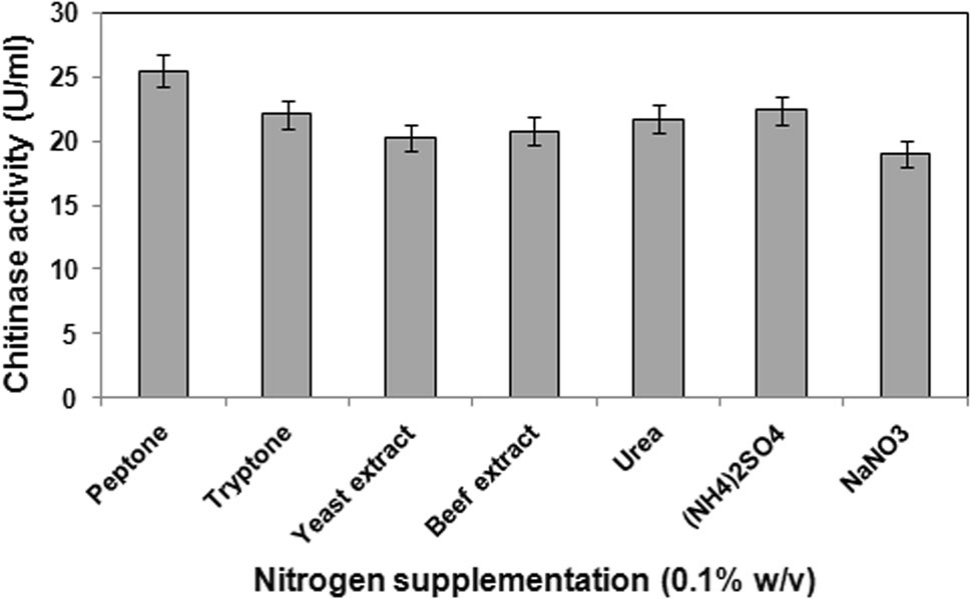
Effect of nitrogen supplements on chitinase production by *B. pumilus* JUBCH08. Data represent mean ± SD (*n* = 3); *P* < 0.05

Among the different organic and inorganic nitrogen sources added to the mineral salt broth, peptone was the most effective nitrogenous additive resulting in highest chitinase production (25.5 U/ml). Ammonium sulfate acted as the best inorganic nitrogen source (Fig. 1).

In general, chitinase production was improved by addition of organic nitrogen than inorganic nitrogen. This might be due to the fact that organic nitrogen sources contain most types of amino acids and growth factors vital for bacterial growth and could be metabolized directly by the cells, consequently promoting chitinase production. However, the present finding also indicates that ammonium sulfate acting as an inorganic nitrogen source may be used for large scale chitinase production as an alternative to peptone.

Wang et al. (2001) reported that peptone (0.1 % w/v) induced high levels of chitinase from *B.* *cereus.* Incorporation of different organic and inorganic nitrogen sources in the media resulted in maximum chitinase production by *B. cereus* JF68 in the presence of peptone (Jabeen and Qazi 2014).

### Effect of initial pH of the medium

**Fig. 2:**
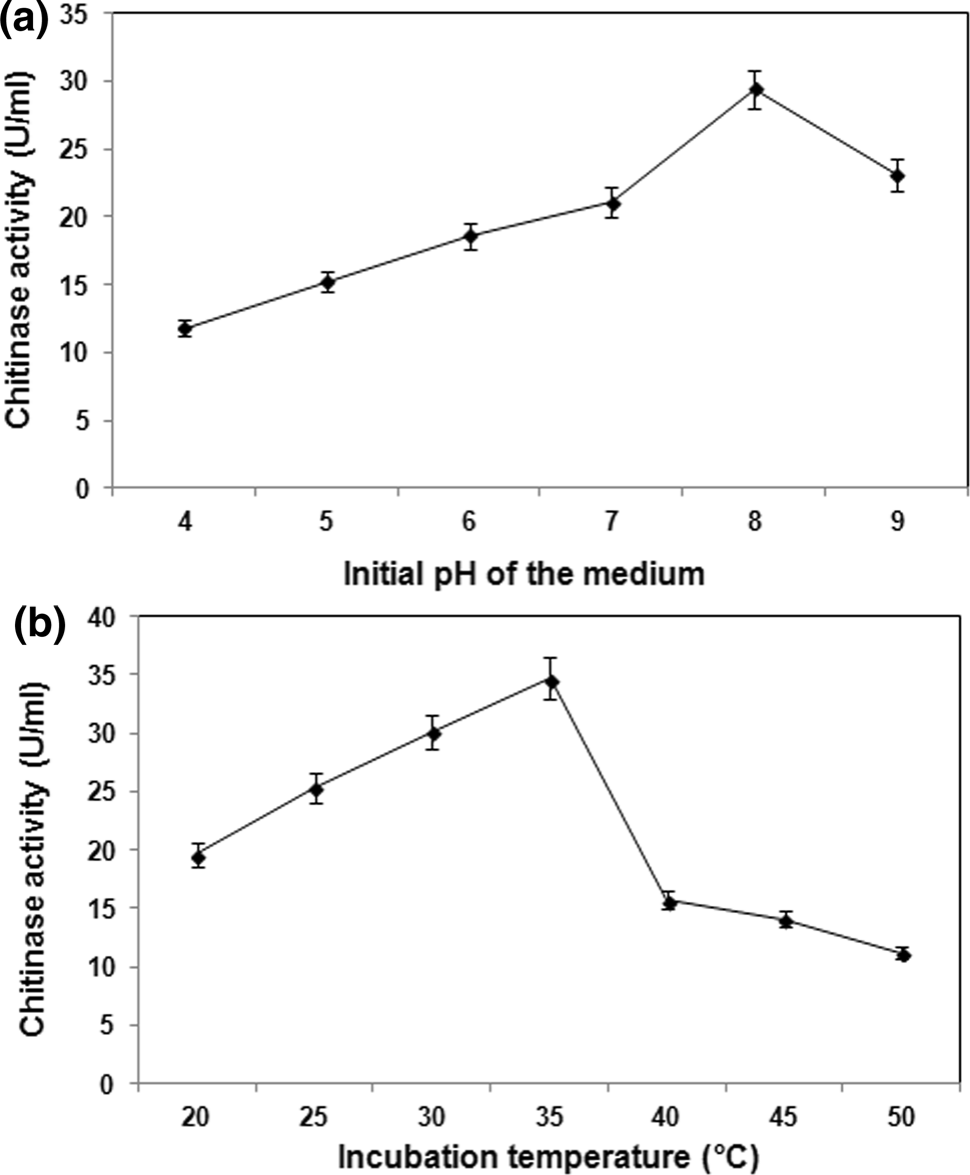
Effect of physical parameters on chitinase production by *B. pumilus* JUBCH08. **a** Chitinase activity under the influence of initial pH of the medium. **b** Chitinase activity at different incubation temperatures. Data represent mean ± SD (*n* = 3); *P* < 0.05

The pH of the immediate external environment partially governs the cytoplasmic pH and is believed to affect the rate of enzyme mediated reaction and structure of many biological molecules including proteins and nucleic acids. The present study reports appreciable chitinase production between pH 6.0 and 9.0, with the maximum (29.4 U/ml) recorded at pH 8.0. Decrease in enzyme yield was observed under acidic conditions (Fig. 2a).

The probable reason for maximum production of chitinase at pH 8.0 by *B. pumilus* JUBCH08 could be because most microbial extracellular enzymes are best produced at pH supporting maximum enzyme activity (Mc Tigue et al. 1994). The present observation is in corroboration with an earlier report where chitinase production by *B. pabuli* K1 was optimum at pH 8.0 (Frändberg and Schnürer 1994).

### Effect of incubation temperature

In bioprocesses, specific temperature requirement and its regulation are the most critical parameters because cultivation temperature affects protein synthesis by influencing the rate of biochemical reactions within the microbial cell and thereby consequently inducing or repressing enzyme production (Nascimento and Martins 2004).

When *B. pumilus* JUBCH08 was grown at 20–50 °C, chitinase production was maximum (34.7 U/ml) at 35 °C. The enzyme production decreased above 40 °C (Fig. 2b). This result indicated the organism’s mesophilic preference for enzyme production. The present observation regarding the effect of incubation temperature on chitinase production is in complete agreement with a previous finding that reported 35 °C as the optimum temperature for chitinase production by *B. amyloliquefaciens* SM3 strain (Das et al. 2012).

### Effect of incubation time

The chitinase yield was initially low and after a lag phase of approximately 48 h, the yield gradually increased. Maximum chitinase production (39.3 U/ml) was recorded at 72 h of incubation, beyond which the chitinase production reduced rapidly.

From the present observation, it may be anticipated that the initial linear nature of chitinase production by *B. pumilus* JUBCH08 was probably due to the fact that chitin being a hard-decomposable compound, longer time was needed by the bacterial culture to adapt to this substrate to produce chitinase. Till 72 h of incubation, *N*-acetyl glucosamine liberated by the chitinase activity was possibly used by *B. pumilus* JUBCH08 as a carbon and nitrogen source. Beyond 72 h, greater amount of accumulated *N*-acetyl glucosamine might have hampered further production of the enzyme by a feedback mechanism. The other possible reason for the decrease in chitinase yield might be due to activation of proteinase mediated degradation or inactivation of the chitinase by unclear mechanisms (Donderski and Trzebiatowska 2000).

### Purification profile of chitinase

As summarized in Table 1, the chitinase from *B. pumilus* JUBCH08 was purified by the combination of several purification steps. The chitinase was purified to 25.1-fold in three steps with a recovery of 74.7 %. We relate the decrease in total chitinase activity to the loss of enzyme during the purification steps.

**Table 1:** Purification summary of chitinase produced by *B. pumilus* JUBCH08

Steps	Activity (U/ml)	Protein content (mg)	Specific activity (U/mg)	Recovery (%)	Purification fold
Crude enzyme	40.34	390	0.10	100	1.0
Ammonium sulfate desalted	37.45	170	0.22	92.8	2.2
Sephade× G-200	35.16	29	1.21	87.1	12.1
DEAE-sepharose	30.17	12	2.51	74.7	25.1

### Effect of pH on chitinase activity and stability

**Fig. 3:**
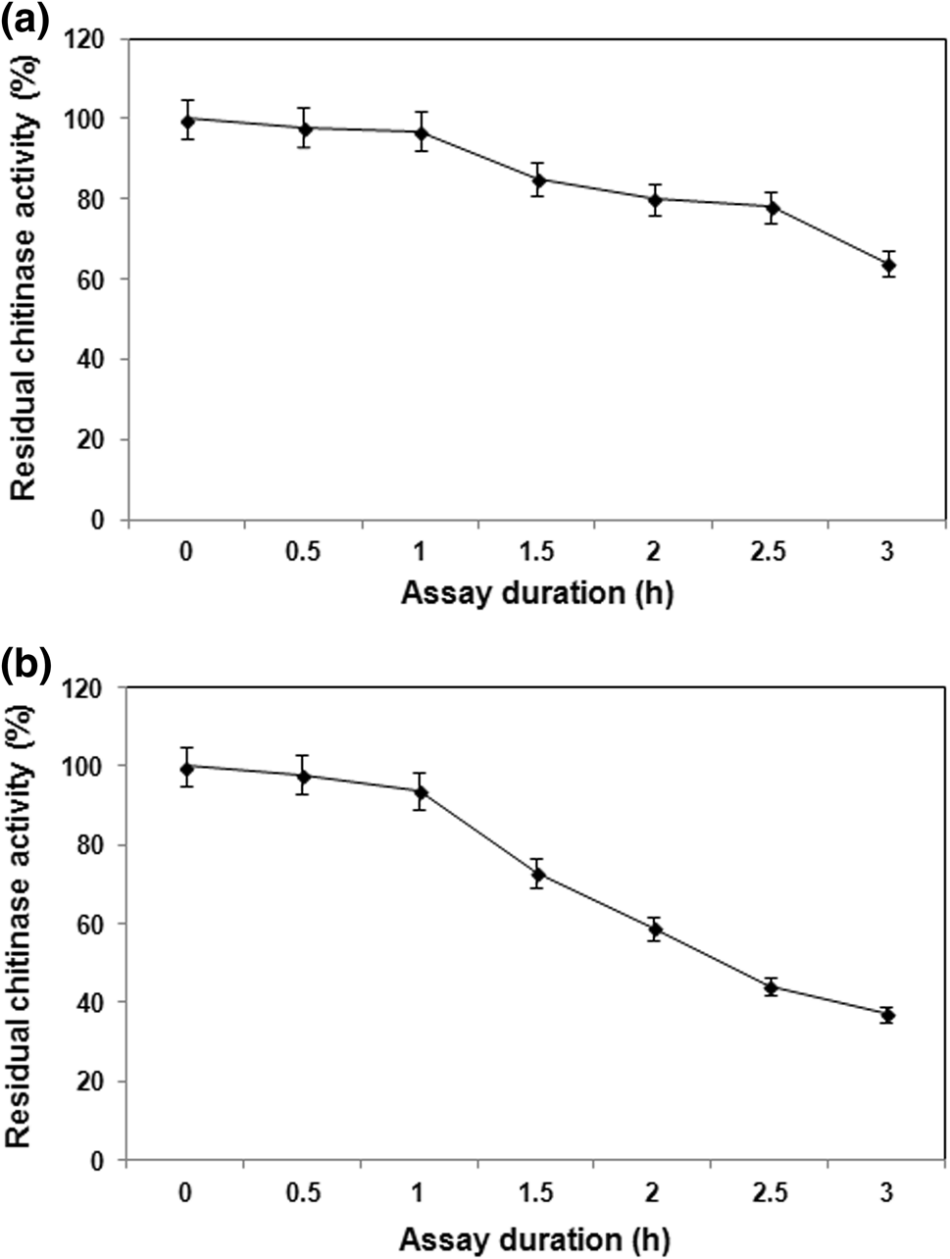
Residual chitinase activity at different time intervals. **a** Activity at pH 8.0. **b** Activity at 70 °C. Data represent mean ± SD (*n* = 3); *P* < 0.05

The chitinase from *B. pumilus* JUBCH08 showed better enzyme activity at alkaline pH range as compared to the acidic pH range, wherein pH 8.0 supported maximum enzyme activity. At pH 8.0, the chitinase retained approximately 97 and 64 % of the original activity for 1 and 3 h, respectively (Fig. 3a).

The chitinase from *B. pumilus* JUBCH08 was different from many other bacterial chitinases in having its pH optima at pH 8.0. Characterization of a novel chitinase from *B. brevis* revealed that the enzymatic activity was stable at pH 6–10 and the enzyme was most active at pH 8.0 (Li et al. 2002). The purified chitinase from *B.* *licheniformis* SK-1 revealed activity optima at pH 6 and 8, respectively, when colloidal chitin was used as substrate (Kudan and Pichyangkura 2009).

### Effect of temperature on chitinase activity and stability

In the present study, the chitinase was stable over a wide range of temperature (50–80 °C) with the maximum activity recorded at 70 °C. The enzyme retained approximately 94 and 37 % of its original activity for 1 h and 3 h, respectively, at 70 °C (Fig. 3b). Similar to the present study, the optimal temperature and pH of the chitinase produced by *B. licheniformis* MB-2 were 70 °C and 6.0, respectively (Toharisman et al. 2005).

### Effect of metal ions on chitinase activity

**Fig. 4:**
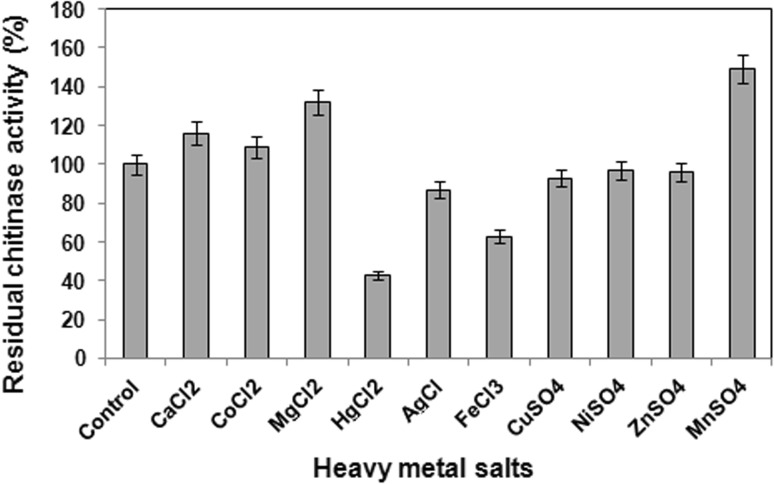
Effect of metal ions on residual chitinase activity. Data represent mean ± SD (*n* = 3); *P* < 0.05

The chitinase activity of *B. pumilus* JUBCH08 increased in the presence of Mg^2+^, Co^2+^, Ca^2+^ and Mn^2+^ ions. Zn^2+^, Ni^2+^ and Cu^2+^ ions slightly inhibited the chitinase activity, whereas Fe^3+^, Ag^+^ and Hg^2+^ proved to be inhibitory (Fig. 4). The result from the present study indicated that metal ions influenced the chitinase activity. This is in agreement with earlier studies wherein the chitinases from *Bacillus* MH-1 and *Bacillus* sp. DAU101 were activated by Mn^2+^ and Co^2+^, respectively (Sakai et al. 1998; Lee et al. 2007).

### Michaelis and rate constant determination

The *K*_*m*_ and *V*_max_ values of the enzyme were 0.13 mg/ml and 38.23 U/ml, respectively. These results are significant as the small *K*_*m*_ value indicates a high affinity of the enzyme toward the substrate (Plalmer 1987).

### Determination of molecular weight

The purified chitinase was a monomer with an apparent molecular weight of 64 kDa. In corroboration to the present finding, chitinase from *B. licheniformis* Mb-2 was found to have a molecular weight of 67 kDa (Toharisman et al. 2005). The molecular mass of the exochitinase from *B.* *thuringiensis* subsp. *pakistani* was 66 kDa (Thamthianku et al. 2001).

### Antagonistic activity against phytopathogenic fungus

**Fig. 5:**
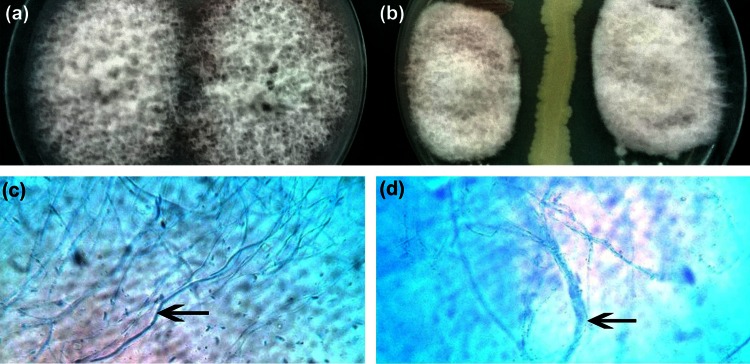
Mycelial inhibition of *F. oxysporum* by *B. pumilus* JUBCH08 in dual culture method, **a** PDA plate inoculated with *F. oxysporum* (control), **b** PDA plate inoculated with *F. oxysporum* and *B. pumilus* JUBCH08. Light microscopic observations of mycelial changes in *F. oxysporum* from the zone of interaction (magnification: 400×), **c** intact mycelium from the control plate, indicated by *black arrow*, **d** swollen and ruptured mycelium from the plate showing zone of inhibition, indicated by *black arrow*

Antagonistic activity of several *Bacillus* species against a number of pathogenic fungi is well documented. By the dual plate method, 45 % inhibition of *F. oxysporum* MTCC 7678 hyphae was observed when cultured in the presence of *B. pumilus* JUBCH08. However, in the control plate, no inhibition of the fungal hyphae was recorded. This antagonistic mechanism is majorly attributed to the action of bacterial chitinase on the fungal cell wall which contains chitin as a major structural component. Microscopic observation of *F. oxysporum* MTCC 7678 mycelia near the zone of inhibition revealed marked morphological changes in hyphae (hyphae appearing swollen, vacuolated, fragmented and distorted) compared to mycelia observed (intact without any distortion) in the control plate (Fig. 5). These morphological changes in the fungal hyphae when cultured in the presence of *B. pumilus* JUBCH08 are evidences of the bacterial chitinase activity on the fungal cell wall (Taechowisan et al. 2003).

Wilt is a widespread plant disease caused by many forms of soil-inhabiting *Fusarium,* especially *F. oxysporum*. Several investigations were undertaken to venture into the biological control of *F. oxysporum* by chitinolytic bacteria. The result of the dual plate assay presented here supports the mechanism of antagonism of *B. pumilus* JUBCH08 against *F. oxysporum* MTCC 7678 mediated through chitinase production. Several studies have demonstrated that bacterial chitinases can cause deformation and inhibition of viable fungal hyphae (Ashwini and Srividya 2014).

## Conclusions

The present investigation revealed that *B. pumilus* JUBCH08 is a potent chitinase producer. Medium supplemented with chitin and peptone, at initial pH 8.0, when incubated at 35 °C for 72 h favored highest chitinase production. The chitinase was unique in some properties, being functional under alkaline conditions and displaying thermostability at 70 °C for 1 h. The results of the dual plate assay emphasized that the chitinolytic bacterium greatly inhibited the fungal pathogen through lytic mechanism, a characteristic that may be utilized for the formulation of a biocontrol agent as an alternative to harmful chemical pesticides.

## References

[CR1] Allison SD, Chacon SS, German DP (2014). Substrate concentration constraints on microbial decomposition. Soil Biol Biochem.

[CR2] Altschul SF, Gish W, Miller W, Myers EW, Lipman DJ (1990). Basic local alignment search tool. J Mol Biol.

[CR3] Ashwini N, Srividya S (2014). Potentiality of *Bacillus subtilis* as biocontrol agent for management of anthracnose disease of chilli caused by *Colletotrichum gloeosporioides* OGC1. 3. Biotech.

[CR4] Chaiharn M, Lumyong S, Hasan N, Plikomol A (2013). Solid-state cultivation of *Bacillus thuringiensis* R 176 with shrimp shells and rice straw as a substrate for chitinase production. Ann Microbiol.

[CR5] Collins DP, Jacobsen BJ, Maxwell B (2003). Spatial and temperal population dynamics of a phyllosphere colonizing *Bacillus subtilis* biological control agent of sugar beet cercospora leaf spot. Biol Control.

[CR6] Das MP, Rebecca JL, Sharmila S, Banerjee A, Kumar D (2012). Identification and optimization of cultural conditions for chitinase production by *Bacillus amyloliquefaciens* SM3. J Chem Pharm Res.

[CR7] Donderski W, Trzebiatowska M (2000). Influence of physical and chemical factors on the activity of chitinases produced by planktonic bacteria isolated from Jeziorak Lake. Pol J Environ Stud.

[CR8] Frändberg E, Schnürer J (1994). Chitinolytic properties of *Bacillus pabuli* K1. J Appl Bacteriol.

[CR9] Huang CJ, Wang TK, Chung SC, Chen CY (2005). Identification of an antifungal chitinase from a potential biocontrol agent, *Bacillus**cereus*. J Biochem Mol Biol Sci.

[CR10] Jabeen F, Qazi JI (2014). Isolation of chitinase yielding *Bacillus cereus* JF68 from soil employing an edible crab shell chitin. J Sci Ind Res.

[CR11] Kudan S, Pichyangkura R (2009). Purification and characterization of thermostable chitinase from *Bacillus licheniformis* SK-1. Appl Biochem Biotechnol.

[CR12] Kuddus SM, Ahmad RIZ (2013). Isolation of novel chitinolytic bacteria and production optimization of extracellular chitinase. J Genet Eng Biotechnol.

[CR13] Laemmli UK (1970). Cleavage of structural proteins during the assembly of the head of bacteriophage T4. Nature.

[CR14] Lee YS, Park IH, Yoo JS, Chung SY, Lee YC, Cho YS, Ahn SC, Kim CM, Choi YL (2007). Cloning, purification, and characterization of chitinase from *Bacillus* sp. DAU101. Bioresour Technol.

[CR15] Li S, Zhao ZA, Li M, Gu ZR, Bai C, Huang WD (2002). Purification and characterization of a novel chitinase from *Bacillus brevis*. Sheng Wu Hua Xue Yu Sheng Wu Wu Li Xue Bao (Shanghai).

[CR16] Lien TS, Yu ST, Wu ST, Too JR (2007). Induction and purification of a thermophilic chitinase produced by *Aeromonas* sp. DYU-too7 using glucosamine. Biotechnol Bioproc Eng.

[CR17] Lineweaver H, Burk D (1934). The determination of enzyme dissociation constants. J Am Chem Soc.

[CR18] Lowry OH, Rosebrough NJ, Farr AL, Randall RJ (1951). Protein estimation with the folin phenol reagent. J Biol Chem.

[CR19] Mc Tigue MA, Kelly CT, Fogarty WM, Doyle EM (1994). Production studies on the alkaline amylase of three alkalophilic *Bacillus* spp. Biotechnol Lett.

[CR20] Miller GL (1959). Use of dinitrosalicylic acid reagent for determination of reducing sugar. Anal Chem.

[CR21] Nascimento WCA, Martins MLL (2004). Production and properties of an extracellular protease from thermophilic *Bacillus sp*. Braz J Microbiol.

[CR23] Sakai K, Yokota A, Kurokawa H, Wakayama M, Moriguchi M (1998). Purification and characterization of three thermostable endochitinases of a noble *Bacillus* strain, MH-1, isolated from chitin-containing compost. Appl Environ Microbiol.

[CR24] Swiontek Brzezinska M, Jankiewicz U, Burkowska A, Walczak M (2014). Chitinolytic microorganisms and their possible application in environmental protection. Curr Microbiol.

[CR25] Taechowisan T, Peberdy JF, Lumyong S (2003). Chitinase production by endophytic *Streptomyces aureofaciens* CMUAc130 and its antagonism against phytopathogenic fungi. Ann Microbiol.

[CR27] Toharisman A, Suhartono MT, Spindler-Barth M, Hwang J, Pyun Y (2005). Purification and characterization of a thermostable chitinase from *Bacillus licheniformis* Mb-2. World J Microb Biot.

[CR28] Wang SL, Chang WT (1997). Purification and characterization of two bifunctional chitinases/lysozymes extracellularly produced by *Pseudomonas aeruginosa* K-187 in shrimp and crab shell powder medium. Appl Environ Microbiol.

[CR29] Wang SY, Moyne AL, Thottappilly G, Wu SJ, Locy RD, Singh NK (2001). Purification and characterization of a *Bacillus cereus* exochitinase. Enzyme Microb Technol.

[CR30] Wang SL, Hsiao WJ, Chang WT (2002). Purification and characterization of an antimicrobial chitinase extracellularly produced by *Monascus purpureus* CCRC31499 in a shrimp and crab shell powder medium. J Agric Food Chem.

[CR31] Zhu X, Zhou Y, Feng J (2007). Analysis of both chitinase and chitosanase produced by *Sphingomonas* sp. CJ-5. J Zhejiang Univ Sci B.

